# An inversion model for estimating the negative air ion concentration using MODIS images of the Daxing’anling region

**DOI:** 10.1371/journal.pone.0242554

**Published:** 2020-11-24

**Authors:** Cui Yue, Zhao Yuxin, Zhang Nan, Zhang Dongyou, Yang Jiangning

**Affiliations:** Heilongjiang Province Key Laboratory of Geographical Environment Monitoring and Spatial Information Service in Cold Regions, Harbin Normal University, Harbin, China; Northeastern University (Shenyang China), CHINA

## Abstract

The negative air ion (NAI) concentration is an essential indicator of air quality and atmospheric pollution. The NAI concentration can be used to monitor air quality on a regional scale and is commonly determined using field measurements. However, obtaining these measurements is time-consuming. In this paper, the relationship between remotely sensed surface parameters (such as land surface temperature, normalized difference vegetation index (NDVI), and leaf area index) obtained from MODIS data products and the measured NAI concentration using a stepwise regression method was analyzed to estimate the spatial distribution of the NAI concentration and verify the precision. The results indicated that the NAI concentration had a negative correlation with temperature, leaf area index (LAI), and gross primary production while it exhibited a positive correlation with the NDVI. The relationship between land surface temperature and the NAI concentration in the Daxing’anling region is expressed by the regression equation of y = -35.51x_1_ + 11206.813 (R^2^ = 0.6123). Additionally, the NAI concentration in northwest regions with high forest coverage was higher than that in southeast regions with low forest coverage, suggesting that forests influence the air quality and reduce the impact of environmental pollution. The proposed inversion model is suitable for evaluating the air quality in Daxing’anling and provides a reference for air quality evaluation in other areas. In the future, we will expand the quantity and distribution range of sampling points, conduct continuous observations of NAI concentrations and environmental parameters in the research areas with different land-use types, and further improve the accuracy of inversion results to analyze the spatiotemporal dynamic changes in NAI concentration and explore the possibility of expanding the application areas of NAI monitoring.

## Introduction

Negative air ions (NAI) are negatively charged atoms or molecules in the atmosphere. NAI mainly consist of negative oxygen ions because the “capture” ability of oxygen is stronger than that of other air molecules, and oxygen has easy access to the free electrons of the NAI [1]. NAI in nature are generally produced by ionization, the Lenard effect, the point discharge of plant tips, the photoelectric effect, and other atmospheric discharge phenomena such as lighting and thunderstorms; the effective method of artificially generating NAI is corona discharge [2].

NAI can improve the physiology of people and maintain their health at certain levels of concentration [3]. At NAI concentrations of more than 1,000 ind/cm^3^, beneficial health effects have been observed; and at NAI concentration of 8,000 ind/cm^3^, diseases have been cured [4–7]. In contrast, at NAI concentrations of less than 40~50 ind/cm^3^, people may experience headaches and insomnia. High NAI concentrations can relieve depression, improve basic information processing capacity, as well as prevent and cure cognition impairment [8–10]. Besides, negative oxygen ions can improve people’s sleep quality and blood circulation, enhance cardiopulmonary functions, aerobic metabolism, and recovery after movements, as well as cure pneumoconiosis by integrating with low load aerobic Yoga [11–15]. An NAI generator can eliminate particulate matters in the atmosphere while filtering and inactivating aerosolized viruses [16–19]. Therefore, an investigation of the NAI concentration is of great significance for human physical and mental health and the improvement of air quality.

NAI research has become an essential topic in recent years. International research on air anions has primarily focused on the effects of air anions on biological organisms, clinical medical applications, spatiotemporal distribution and air quality evaluation [20–23]. The spatial and temporal variations of NAI and their influencing factors have attracted much attention [24]. Studies have revealed that NAI concentrations depended mainly on location, vegetation characteristics, climate, and meteorological conditions. For example, NAI concentrations were demonstrated to be higher in summer than in winter [25, 26]. With the premise of controlling other variables, the NAI concentration could be improved by increasing the number of plants and exerting pulsed electric fields (PEF) on vegetation [27–29]. From the perspective of area research, the NAI concentration of falls and forests is generally higher than that of greenbelts and bare lands [30, 31]. Since most research on NAI concentrations in China was conducted in southern and coastal areas, the results may not apply to northern China, and the traditional anion concentration study is generally in a limited area. During the past few years, research activity on this topic has been increased overseas while these studies have emphasized small areas and conducted qualitative analyses; there is little research on the temporal and spatial distribution of NAI and the retrieval of NAI with inversion models [25]. During the practical application processes, it is difficult for researchers to grasp the spatial distribution of NAI concentration; obstructing some works such as air quality monitoring, health care, and urban planning. The innovation of our paper is to use remote sensing data to study the anion concentration in a large area.

In this study, an inversion model of the NAI concentration was developed using remotely sensed data of the Daxing’anling region. Forty-four sample points were adopted in different land cover types. Besides, field measurements of the NAI concentration were conducted in the summer of 2016. Afterward, the key environmental factors affecting the NAI concentration were determined, and a relationship between these factors and the NAI concentration was developed. Finally, an evaluation of the above research results was performed, and the utilization potentiality and scientific values of NAI concentration inversion in future researches were discussed.

## Methods and materials

### Study area

**Fig 1:**
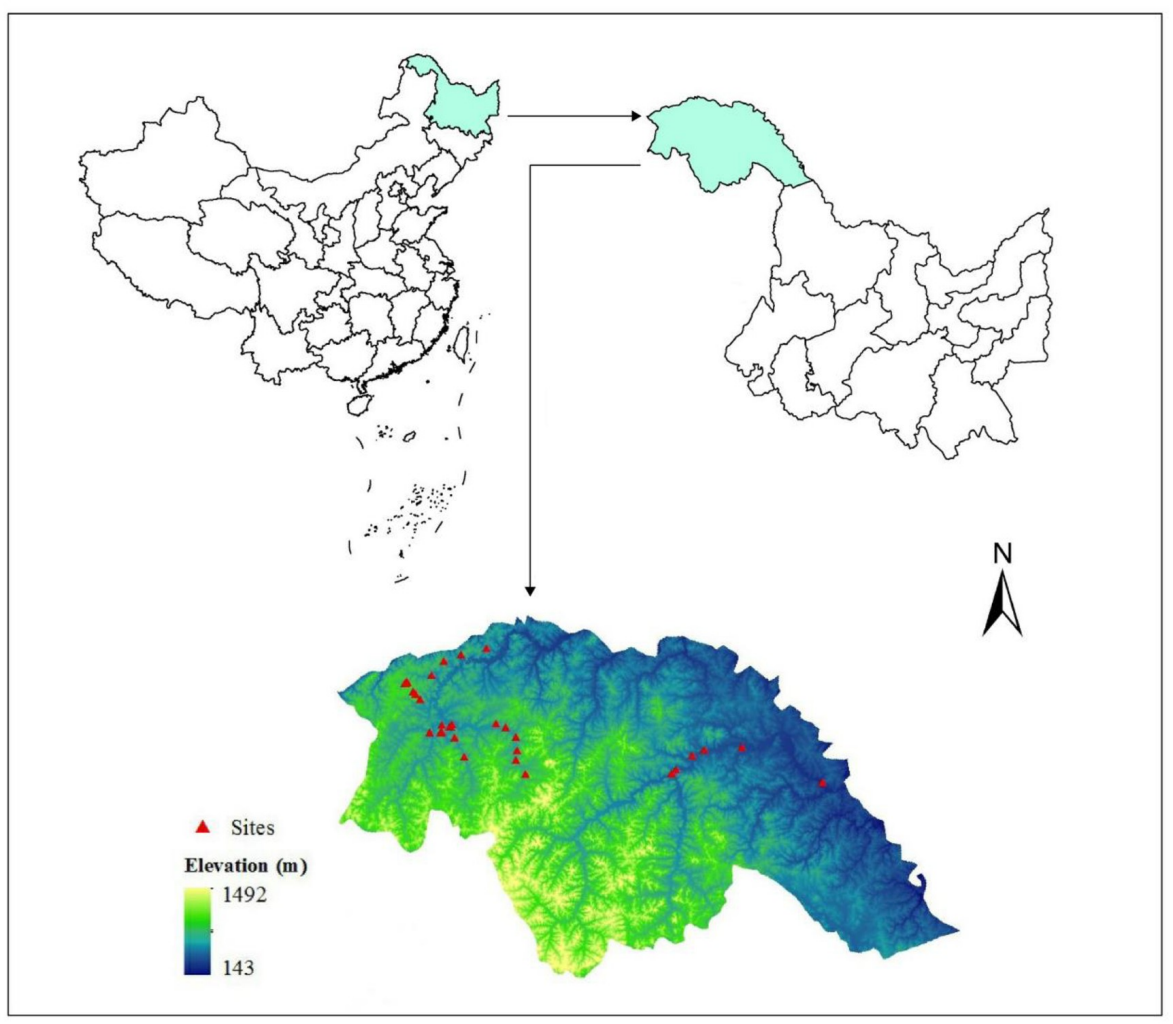
Location of the study area.

The Daxing’anling region (50°08' -53°34' N, 121°11'-127°00' E) is located in the north of Heilongjiang province and northeast of Inner Mongolia; the area is the largest and northernmost forest region in China, significant to forestry [32]. The Daxing’anling region covers 64,800 km^2^; high-elevation areas occur in the west; low-elevation areas occur in the east with gentle terrain slopes (Fig 1). The Daxing’anling region has dense virgin forests, and the dominant trees are Xingan larch, camphor pine, red spruce, white birch, Mongolian oak, and aspen.

### Data source and processing

#### Field sampling of NAI concentration

Field sampling was conducted in late July of 2016 due to active vegetation growth and peak photosynthesis in the summer. The samples were obtained in five land use/landcover types: forest land, grassland, cultivated land, buildings, and water (S1 Table). The predominant types of land cover were grassland and woodland. With the purpose of controlling sampling time, reducing plant physiological activities and surface temperature changes caused by seasonal factors, and improving data acquisition efficiency, sampling points in this research were laid along the roads with an equal interval as far as possible and a distance of at least 1 km from the main roads to avoid the influence of passing vehicles on the NAI concentration [33].

GPS measurements of latitude and longitude were collected at each site; the negative and positive ion concentrations at 150 cm above the ground were obtained using an air ion counter (COM-3200-PRO) on a tripod. Operators should keep a distance of 2~3m from the equipment while measuring to record the reading after it was stabilized, with an error of ≤±5%.

All measurements were obtained under clear and calm weather conditions and at least 50 m inside the forest canopy to minimize the influence of weather, fog, and environmental pollution.

#### MODIS images

In this study, MODIS data were used to develop the inversion model of the NAI concentration. The data were downloaded from the United States Geological Service (USGS) website (http://glovis.usgs.gov). Four data products (MOD11A2, MOD13A3, MOD15A2, and MOD17A2) for July 2016 were employed to extract LST, NDVI, LAI, and GPP, respectively.

### Study method

Data acquired through remote sensing technology is of an outstanding space-time coverage and continuity, making it suitable for monitoring vegetation indexes and climate conditions [34, 35]. Vegetational covers and climate changes can be broadly, simply, and efficiently measured through vegetation index; this may have certain correlations with the formation of NAI in nature while no relevant literature has been found up to now. In this paper, the relationships between remote sensing surface parameters (land surface temperature, normalized difference vegetation index (NDVI), leaf area index, etc.) were firstly analyzed by MODIS data products; then, the spatial distribution and accuracy of the negative ion concentration were estimated by correlation analysis and stepwise regression analysis.

#### Environmental parameters

**Fig 2:**
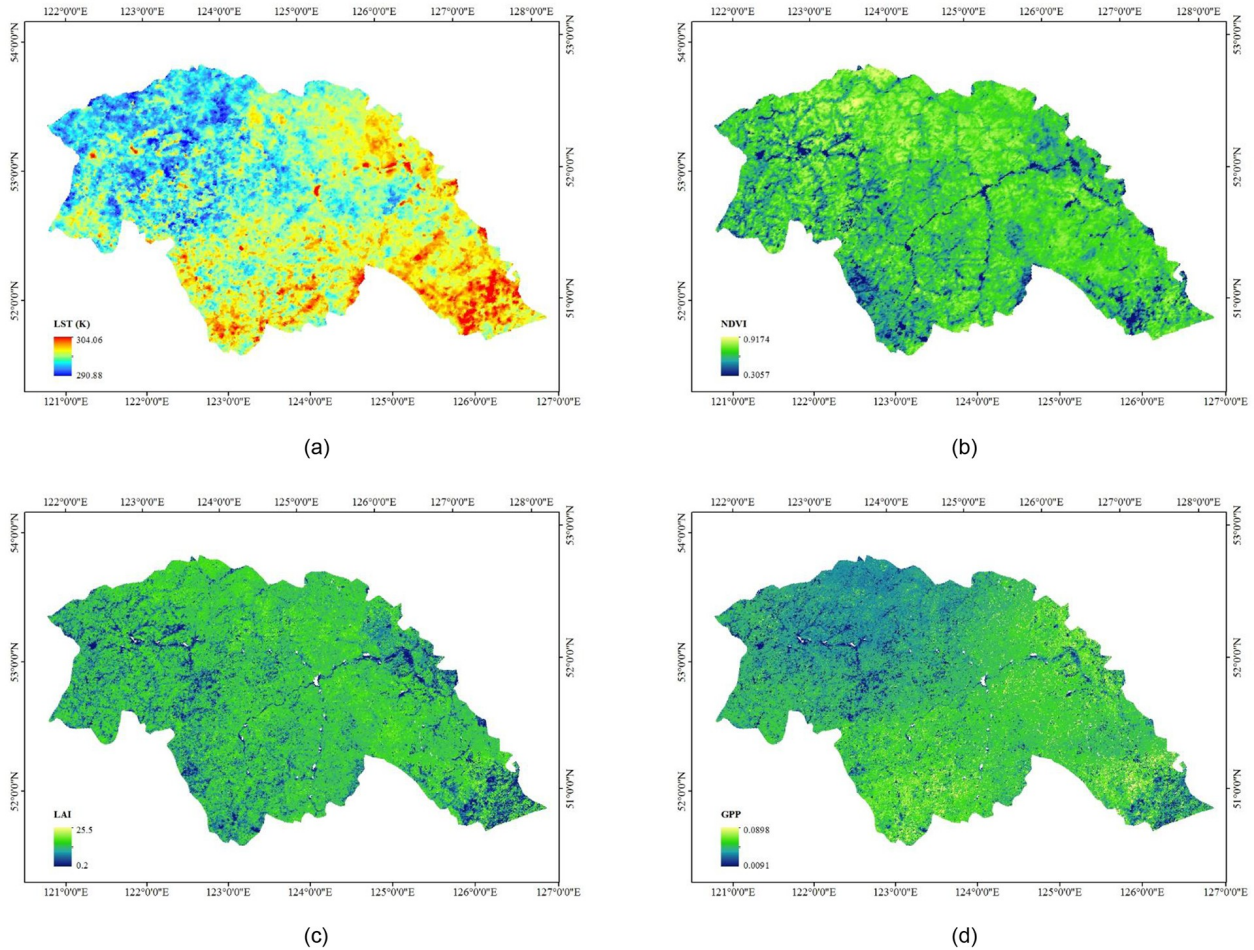
MODIS-based LST (a), NDVI (b), LAI (c), and GPP (d) of the Daxing’anling region.

The MODIS reprojection tool (MRT) was used to convert the HDF format to the TIF format and reproject and resample the data. Besides, ArcGIS 10.1 was adopted to crop the MODIS image to the Daxing’anling region and obtain a TIF format image. Similarly, TIF raster images of the land surface temperature (LST), normalized difference vegetation index (NDVI = (NIR - R)/(NIR + R), NIR: Near-infrared reflectance, R: Red band reflectance), leaf area index (LAI = total leaf area/land area), and gross primary production (GPP: an indicator of vegetation growth) were obtained. Particularly, the clipped MODIS images were filtered with a 3×3 mean filter to smooth the image before the ground scene information was extracted from the data owing to the difference in scale between the MODIS image resolutions and the locations of sampling points that were not located in homogeneous areas. The pixel value within the whole window was balanced through this method, contributing to more accurately reflecting the environmental characteristics of the sampling points and avoiding accidental errors. The mapping results are illustrated in Fig 2.

The vector file with the sample locations and the LST, NDVI, LAI, and GPP raster files were in the same projection to ensure that the data for the correct location were extracted from the raster files. The numerical results are presented in Table 1.

**Table 1:** Values of the four parameters (GPP, LAI, LST, and NDVI) at the sampling points.

Sample point	GPP	LAI	LST	NDVI	Sample point	GPP	LAI	LST	NDVI
**1**	0.11	3.44	291.96	0.87	23	0.11	4.76	295.70	0.84
**2**	0.12	4.03	292.40	0.86	24	0.12	4.16	295.73	0.80
**3**	0.10	2.51	293.05	0.79	25	0.12	4.16	295.73	0.80
**4**	0.11	3.00	293.72	0.84	26	0.11	4.80	295.92	0.87
**5**	0.11	4.30	293.87	0.84	27	0.11	3.01	295.97	0.80
**6**	0.08	1.12	293.91	0.81	28	0.12	4.41	296.09	0.85
**7**	0.11	3.56	294.16	0.83	29	0.12	4.41	296.09	0.85
**8**	0.11	3.67	294.19	0.83	30	0.10	3.28	296.63	0.80
**9**	0.11	3.53	294.20	0.82	31	0.11	3.69	296.63	0.77
**10**	0.11	3.53	294.20	0.82	32	0.11	3.69	296.63	0.77
**11**	0.10	2.97	294.31	0.82	33	0.11	3.69	296.63	0.77
**12**	0.13	5.16	294.39	0.88	34	0.11	3.69	296.63	0.77
**13**	0.11	3.96	294.74	0.80	35	0.11	3.69	296.63	0.77
**14**	0.11	3.79	294.91	0.83	36	0.11	3.69	296.63	0.77
**15**	0.11	3.79	294.91	0.83	37	0.11	3.69	296.63	0.77
**16**	0.12	4.09	295.12	0.82	38	0.09	2.13	297.42	0.69
**17**	0.13	4.69	295.16	0.86	39	0.09	2.13	297.42	0.69
**18**	0.13	4.69	295.16	0.86	40	0.09	2.13	297.42	0.69
**19**	0.12	4.04	295.32	0.85	41	0.10	2.86	298.47	0.71
**20**	0.11	3.26	295.32	0.76	42	0.10	2.86	298.47	0.71
**21**	0.11	3.26	295.32	0.76	43	1.86	14.49	298.53	0.48
**22**	0.11	4.33	295.58	0.84	44	1.86	14.49	298.53	0.48

The LST was retrieved using the equation *NEW*_*DN* = *OLD*_DN*0.02, where OLD_DN denotes the gray value of a pixel of MOD11A2, and NEW_DN represents brightness temperature in Kelvin.

#### Correlation and regression analysis

Correlation coefficient *r* between the parameter values calculated by the SPSS software and the measured NAI concentrations is expressed in Formula 1.

Regression analysis refers to a statistical analysis method used to determine the quantitative relationship between two or more variables. In this study, the regression analysis of the relationship between the NAI concentration and the environmental parameters was conducted using SPSS, and the linear regression models was acquired through a backward regression method. Through backward regression, all environmental parameters were first selected into an inversion regression equation; then, full consideration was given to the combination effect of independent variables; next, those variables with an indistinctive influence on the regression equation were eliminated in order; finally, an inversion model was established and verified by the RMSE (Eqs 2 and 3).

$$r=\frac{\sum_{i=1}^{n}\left(x_{i}-\bar{x})(y_{i}-\bar{y}\right)}{\sqrt{\sum_{i=1}^{n}{\left(x_{i}-\bar{x}\right)}^{2}\sum_{i=1}^{n}{\left(y_{i}-\bar{y}\right)}^{2}}}$$ (1)

$$e_{i}=x_{i}-x_{i}^{\prime }(i=1,2,\cdots ,n)$$ (2)

$$RMSE=\sqrt{\frac{1}{n}\sum_{i=1}^{n}e_{i}^{2}}$$ (3)

where *n* denotes the number of sampling points; *x*_*i*_ and *y*_i_ are measured value of NAI concentration and environmental parameter at the *i*^th^ sampling point (i.e. extracted value of LST, NDVI, LAI, and GPP), respectively; $\bar{x}$ and $\bar{y}$ refer to the mean values of NAI concentration and environmental parameter, respectively; $x_{i}^{\prime }$ and *e*_*i*_ represents the simulation value at the *i*^*th*^ sampling point and the difference value between this simulation value and the measured value, respectively. The higher the absolute value of correlation coefficient *r*, the higher the correlation between NAI concentration and the above environmental parameter; when *r* is positive, the NAI concentration increases as the above environmental parameter increases, and vice versa. Besides, the lower the RMSE, the smaller the difference between measured and simulated values, and the higher the precision of the inversion model.

## Results

### Comparison of the inversion model

The relevant analysis shows that NAI is significantly negatively related with LST, GPP and LAI respectively (*r* = -0.782, -0.459, -0.461, *P<*0.01), and NAI has a significantly positive correlation with NDVI (*r* = 0.611, *P*<0.01). There was a significant relationship between the selected remotely sensed land surface parameters and the NAI concentration measured at the sampling site.

The results of backward regression are provided in Table 2. The models for LST and LAI had the form of y = -32.132x_1_-6.343x_2_ + 10232.557; the regression model for LST had the form of y = -35.515x_1_ + 11206.813.

**Table 2:** Regression results of the relationship between negative air ion concentration and environmental parameters.

	Model	Unstandardized Coefficients (B)	Standard error of B	Significance level of T (Sig)	95% lower confidence interval of B	95% upper confidence interval of B	Standardized Coefficients (Beta)
**1**	LST	-32.132	4.445	0	-41.109	-23.154	-0.708
	LAI	-6.343	2.872	0.033	-12.143	-0.543	-0.216
	Constant	10232.557					
**2**	LST	-35.515	4.361	0	-44.316	-26.714	-0.782
	Constant	11206.813					

**Fig 3:**
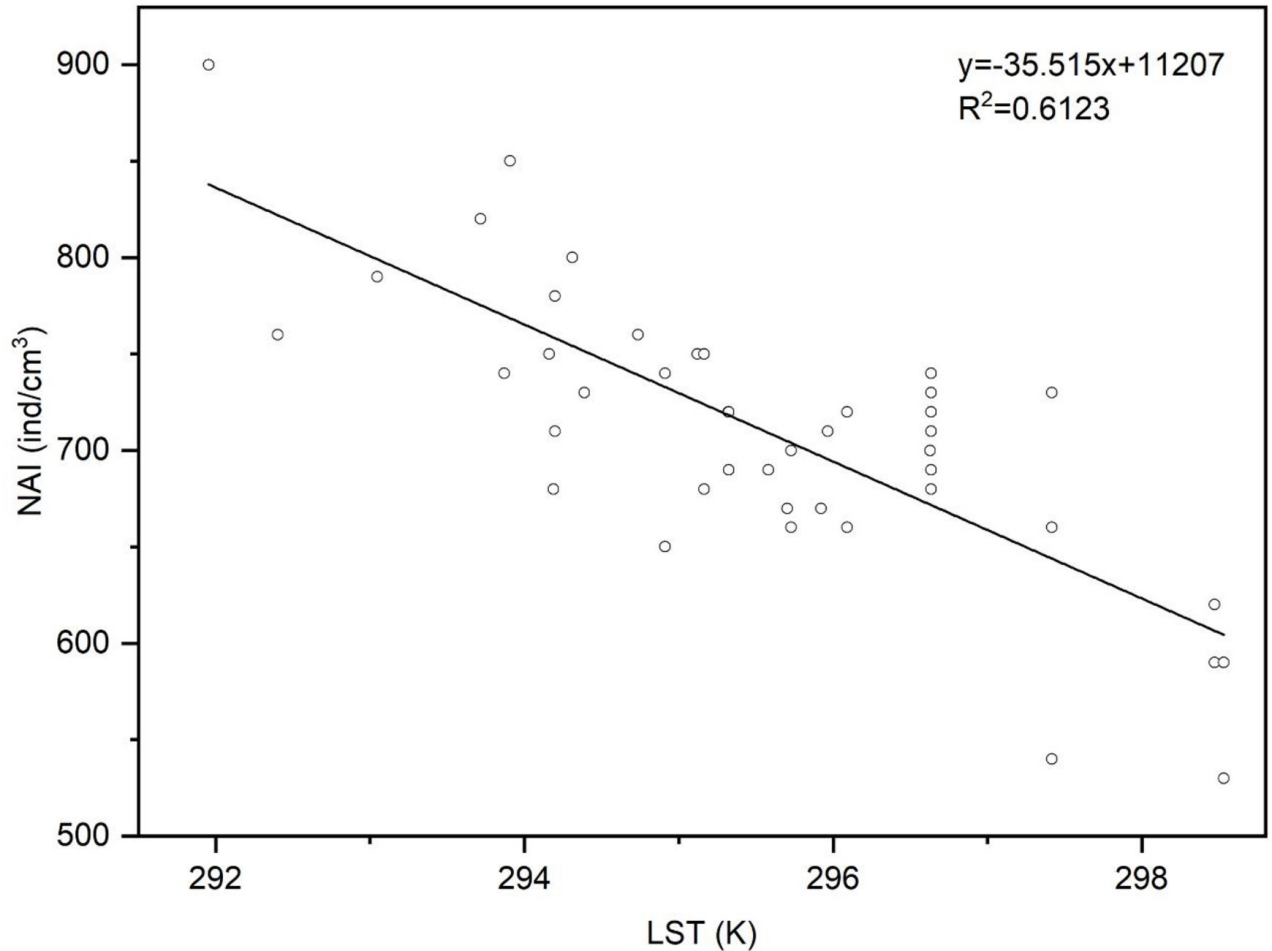
Linear regression of LST and NAI.

The results of the regression analysis illustrate that there is a significant correlation between LST and NAI (Fig 3), while NAI has a significant linear relationship with GPP, LAI and NDVI (S1 Fig). It is concluded that it is feasible to use MODIS images and environmental parameters (LST) to estimate the NAI concentration with an inversion model. The relationship between LST and the NAI concentration in the Daxing’anling region is expressed by the regression equation of y = -35.515x_1_ + 11206.813 (R^2^ = 0.6123).

### Results of the NAI model

The NAI concentrations in the Daxing’anling area in 2013 were obtained, and the LST-NAI regression equations were analyzed. Besides, the measured values and values of the regression equation of 17 points obtained from the same measurement method were compared by performed random sampling. The RMSE is 2.88 ind/cm^3^, suggesting that this method is suitable for the estimation of the NAI concentration [36, 37].

**Fig 4:**
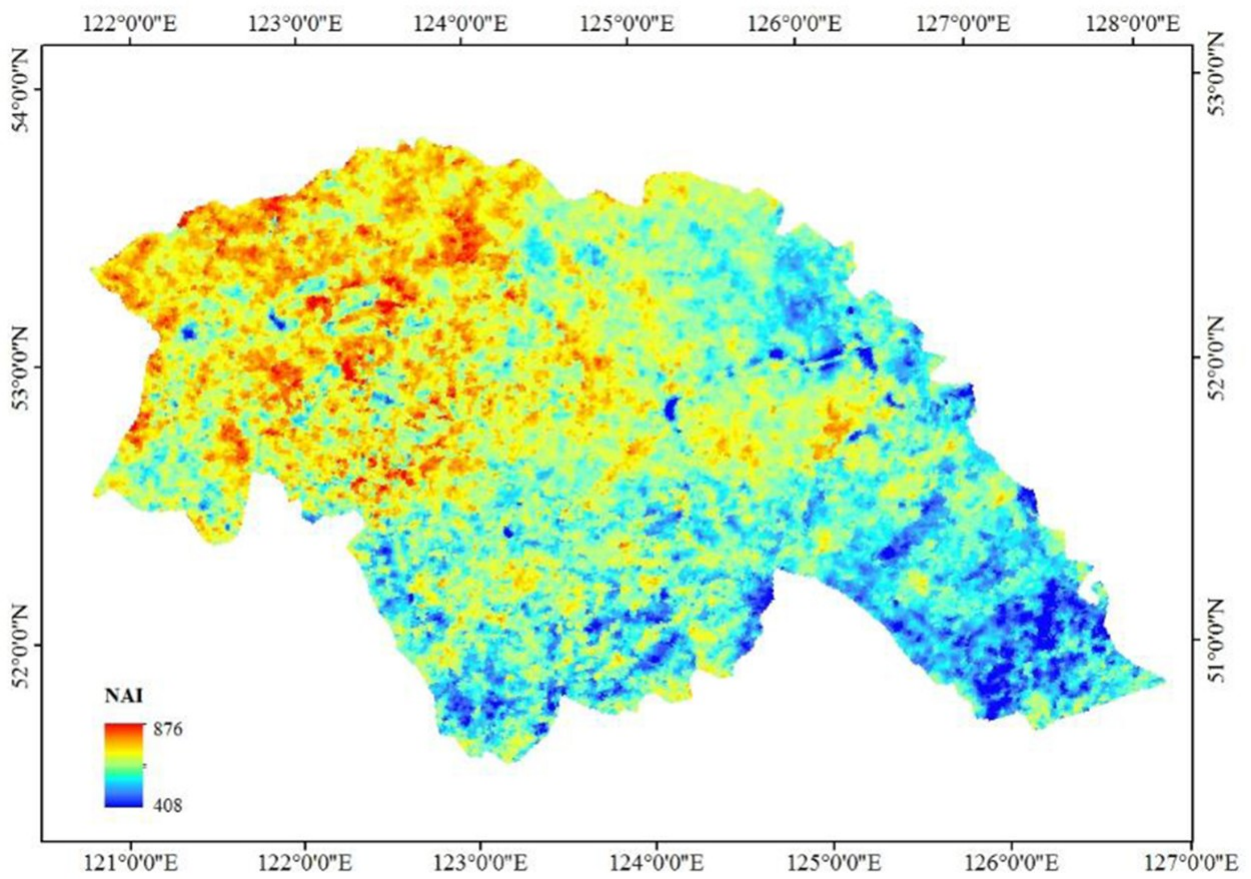
Negative air ion concentration in the Daxing’anling region.

The map of the NAI concentration in the Daxing’anling region based on the regression model is illustrated in Fig 4. The NAI concentration in the Daxing’anling region is within 408~876 ind/cm^3^, exhibiting an obvious spatial difference. Non-forestlands are widely distributed in the southeast part of this region while there are a relatively higher forest coverage rate and fewer human activities in the northwest part; this may be a crucial element leading to the fact that the NAI concentration in the southeast is lower than that in the northwest.

## Discussion

NAI is a valuable resource provided by forests. The concentration of NAI is an important indicator for evaluating the air quality in a region. Negative oxygen ions are also called "air vitamins" and have a positive impact on human health and the ecological environment [38–42]. Researchers have discovered that NAI has a bacteriostatic and sterilizing effect on microorganisms, leading to air purification and improvements in the ecological environment. Besides, the concentration of NAI can be used as an index to monitor urban pollution, and increasing the concentration of NAI can improve air quality and reduce air pollutants and bacterial dust. In recent years, there has been an increasing number of research on NAI monitoring and distribution characteristics, as well as the effect of NAI on the ecological environment. However, most of them are limited to small-scale measurements, focus on the analyses of differences in NAI concentrations of different land-use types and their influence factors, and are deficient in the reports of adopting the 3S technology to invert NAI concentration. With the Daxing’anling region as an example, the vegetation index and LST data extracted from local MODIS products and their correlations with NAI concentration are calculated in this research; meanwhile, the research scope of factors influencing NAI concentration is enlarged, providing beneficial references for an in-depth understanding of generating and consuming mechanisms of NAI. Establishing an inversion model based on environmental factors is a feasible method to grasp the spatial and temporal distribution of NAI and is of great utilization potential.

It is indicated in previous works that it is difficult to make a unified conclusion on the correlation between NAI and meteorological factors because of the complexity of the environment in the research area and non-standardization of monitoring equipment [43]. Most studies have demonstrated that NAI concentrations were positively correlated with temperature [26, 44, 45] while some studies revealed a negative correlation between the NAI concentration and temperature [46, 47]. It was discovered in this research that there was a prominent negative correlation mainly between NAI concentration and LST in the Daxing’anling region; this might be because the photosynthesis in growing seasons was hindered by the ultra-high environmental temperature, resulting in influencing the rate of photoelectric effect of green plants when generating NAI [48]. NAI had a positive correlation with NDVI, suggesting that the NAI concentration level could also be affected by the growth vigor of plants [49]. The regression equation describing the relationship between NAI and LST took the form of y = -35.515x_1_ + 11206.813.

The NAI concentration in the Daxing’anling region is within 408~876 ind/cm^3^, presenting a distribution characteristic that the NAI concentration in the northwest is higher than that in the southeast; this conforms to the local forest distribution. The analysis indicates several reasons for the high NAI concentration in forest land: 1) the soil in forests contains large amounts of radioactive materials that promote the exchange of air in the soil, generate negative oxygen ions, and increase the concentration of NAI; 2) the photosynthesis and transpiration of plants lead to the production of NAI, and the discharge from the leaves of vegetation causes air to ionize and produces negative oxygen ions, resulting in an increase in the concentration of NAI; 3) forest land absorbs large amounts of dust, contributing to reducing the loss of negative ions by the dust and maintaining a constant concentration of NAI.

In this study, only four environmental factors (LST, GPP, LAI, and NDVI) were considered. The field monitoring data were relatively sparse, which may have affected the accuracy of the research results.

## Conclusion

NAI concentrations were measured in the Daxing’anling region in late July of 2016, and MODIS images were obtained during the same period. The relationships between the remotely sensed land surface parameters (LST, NDVI, LAI, and GPP) and the measurements of the NAI concentration were determined using a stepwise regression method to estimate the NAI concentration in the Daxing’anling region. Finally, the spatial distribution of local NAI concentration was inverted. Introducing remote sensing land surface parameters into researches on NAI contributes to more accurately grasping the leading factors influencing NAI concentrations in different regions. Meanwhile, NAI concentration inversion has exhibited a preferable application prospect in several fields, including but not limited to 1) providing references for site selection of forest salutarium improve health care effect of NAI [50]; 2) exploring the changes in NAI distribution during the urbanization process, such as evaluating the influence of urban heat island and water pollution on NAI [51, 52]; 3) scientifically implementing urban planning to promote ecological benefits and human well-being [33]. Besides, the problem of a wide-range grasp of NAI concentration was initially solved by this study. However, this method also exhibits certain limitations due to inconvenient road traffic in the Daxing’anling area, deficient sampling points of NAI, and the comparison of the relationships between only 4 categories of remote sensing surface parameters and NAI concentration. In the future, we will expand sampling density and scope, conduct continuous observations of NAI concentrations and environmental parameters under different land-use types in the research area, improve the inversion model, and enhance inversion accuracy.

## Supporting information

S1 Table The location of the sampling site and the attribute information of the ground objects of the sampling site. (DOCX)

S1 Fig Linear trends of GPP, LAI, NDVI and NAI of sampling site. (DOCX)
