# Supplementary material for: An inversion model for estimating the negative air ion concentration using MODIS images of the Daxing’anling region
Source: PLoS One. 2020 Nov 24;15(11):e0242554. doi: 10.1371/journal.pone.0242554 (PMC7685430; doi:10.1371/journal.pone.0242554)
Supplement: S1 Table — (DOCX) [file pone.0242554.s001.docx]

| Sample point | Longitude（E） | Latitude（N） | Altitude（m） | Land use/landcover types |
| --- | --- | --- | --- | --- |
| 1 | 123.203 | 52.793 | 527 | grassland |
| 2 | 122.646 | 53.365 | 498 | forest land |
| 3 | 123.125 | 52.868 | 495 | cultivated land |
| 4 | 123.185 | 52.706 | 546 | grassland |
| 5 | 122.578 | 52.875 | 479 | forest land |
| 6 | 123.213 | 52.550 | 611 | forest land |
| 7 | 122.484 | 53.297 | 458 | forest land |
| 8 | 123.038 | 52.902 | 518 | forest land |
| 9 | 124.943 | 52.407 | 352 | forest land |
| 10 | 124.942 | 52.407 | 353 | grassland |
| 11 | 123.151 | 52.648 | 551 | water |
| 12 | 122.841 | 53.382 | 562 | forest land |
| 13 | 125.471 | 52.380 | 282 | water |
| 14 | 122.256 | 53.219 | 576 | forest land |
| 15 | 122.258 | 53.220 | 586 | grassland |
| 16 | 122.629 | 52.742 | 498 | grassland |
| 17 | 122.443 | 52.929 | 529 | forest land |
| 18 | 122.443 | 52.929 | 527 | forest land |
| 19 | 122.276 | 53.199 | 547 | forest land |
| 20 | 122.335 | 52.942 | 442 | grassland |
| 21 | 122.335 | 52.943 | 446 | grassland |
| 22 | 122.276 | 53.200 | 534 | forest land |
| 23 | 122.458 | 52.930 | 498 | forest land |
| 24 | 125.084 | 52.424 | 331 | forest land |
| 25 | 125.085 | 52.424 | 321 | forest land |
| 26 | 123.119 | 53.382 | 473 | forest land |
| 27 | 126.181 | 52.030 | 221 | water |
| 28 | 122.312 | 53.161 | 493 | forest land |
| 29 | 122.311 | 53.161 | 503 | forest land |
| 30 | 122.478 | 52.974 | 433 | grassland |
| 31 | 122.218 | 53.282 | 551 | forest land |
| 32 | 122.208 | 53.288 | 552 | grassland |
| 33 | 122.204 | 53.287 | 557 | water |
| 34 | 122.214 | 53.289 | 551 | grassland |
| 35 | 122.216 | 53.289 | 553 | cultivated land |
| 36 | 122.195 | 53.280 | 545 | forest land |
| 37 | 122.193 | 53.280 | 572 | forest land |
| 38 | 122.564 | 52.949 | 457 | grassland |
| 39 | 122.582 | 52.961 | 432 | forest land |
| 40 | 122.549 | 52.951 | 446 | cultivated land |
| 41 | 124.746 | 52.346 | 350 | water |
| 42 | 124.747 | 52.346 | 350 | water |
| 43 | 124.695 | 52.331 | 360 | buildings |
| 44 | 124.696 | 52.331 | 356 | buildings |
