# Supplementary figures and images for: An inversion model for estimating the negative air ion concentration using MODIS images of the Daxing’anling region
Source: PLoS One. 2020 Nov 24;15(11):e0242554. doi: 10.1371/journal.pone.0242554 (PMC7685430; doi:10.1371/journal.pone.0242554)

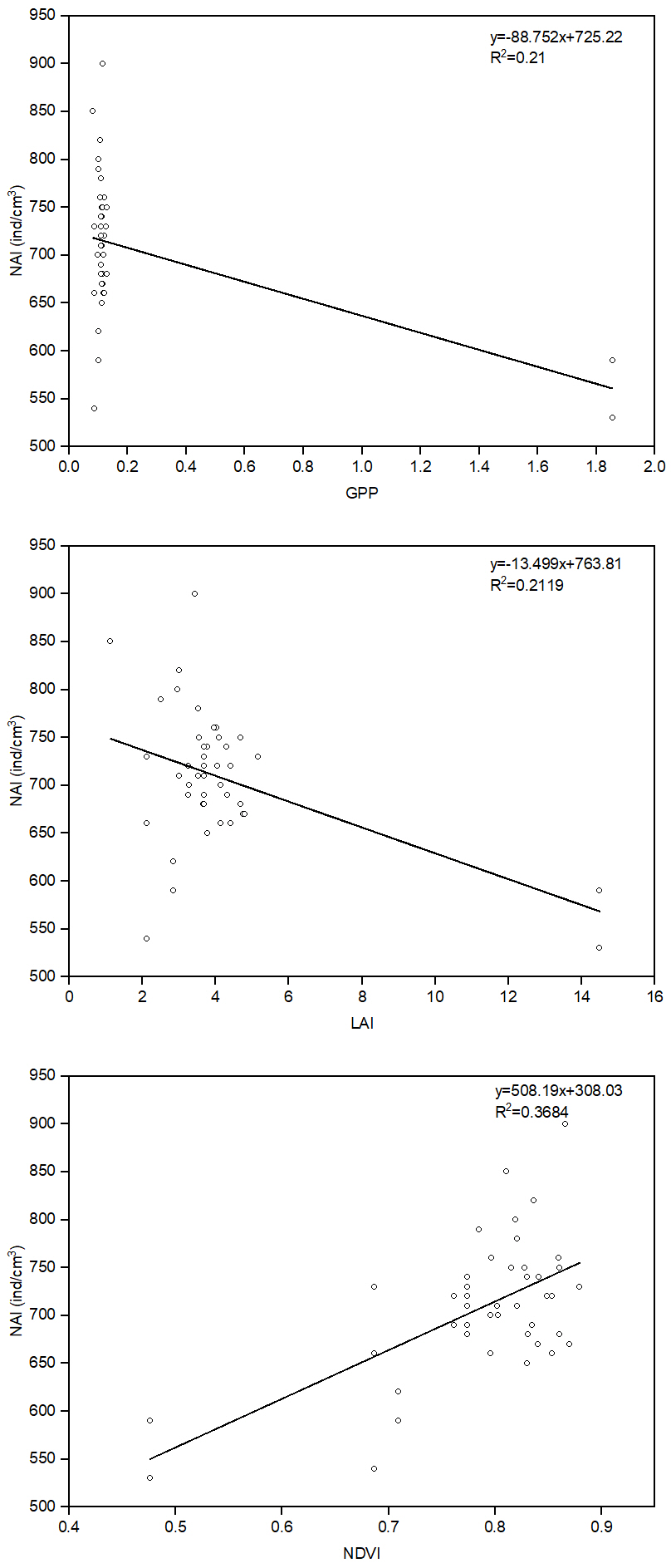

Supplement: S1 Fig — (DOCX) [file pone.0242554.s002.docx]
